# Elevated Levels of Mercapturic Acids of Acrolein and Crotonaldehyde in the Urine of Chinese Women in Singapore Who Regularly Cook at Home

**DOI:** 10.1371/journal.pone.0120023

**Published:** 2015-03-25

**Authors:** Stephen S. Hecht, Woon-Puay Koh, Renwei Wang, Menglan Chen, Steven G. Carmella, Sharon E. Murphy, Jian-Min Yuan

**Affiliations:** 1 Masonic Cancer Center, University of Minnesota, Minneapolis, Minnesota, United States of America; 2 Duke-NUS Graduate Medical School, Singapore, Singapore; 3 Saw Swee Hock School of Public Health, National University of Singapore, Singapore, Singapore; 4 Division of Cancer Control and Population Sciences, University of Pittsburgh Cancer Institute, Pittsburgh, Pennsylvania, United States of America; 5 Department of Epidemiology, Graduate School of Public Health, University of Pittsburgh, Pittsburgh, Pennsylvania, United States of America; Taipei Medical University, TAIWAN

## Abstract

Lung cancer is unusually common among non-smoking women in Southeastern Asia but the causes of this frequently fatal disease are not well understood. Several epidemiology studies indicate that inhalation of fumes from high temperature Chinese style cooking with a wok may be a cause. Only one previous study investigated uptake of potential toxicants and carcinogens by women who cook with a wok. We enrolled three-hundred twenty-eight non-smoking women from Singapore for this study. Each provided a spot urine sample and answered a questionnaire concerning their cooking habits and other factors. The urine samples were analyzed by liquid chromatography-tandem mass spectrometry for mercapturic acid metabolites of acrolein (3-hydroxypropylmercapturic acid), crotonaldehyde (3-hydroxy-1-methylpropylmercapturic acid), and benzene (S-phenylmercapturic acid), accepted biomarkers of uptake of these toxic and carcinogenic compounds. We observed statistically significant effects of wok cooking frequency on levels of 3-hydroxypropylmercapturic acid and 3-hydroxy-1-methylpropylmercapturic acid, but not S-phenylmercapturic acid. Women who cooked greater than 7 times per week had a geometric mean of 2600 (95% CI, 2189-3090) pmol/mg creatinine 3-hydroxypropylmercapturic acid compared to 1901 (95% CI, 1510-2395) pmol/mg creatinine when cooking less than once per week (P for trend 0.018). The corresponding values for 3-hydroxy-1-methylpropylmercapturic acid were 1167 (95% CI, 1022-1332) and 894 (95% CI, 749-1067) pmol/mg creatinine (P for trend 0.008). We conclude that frequent wok cooking leads to elevated exposure to the toxicants acrolein and crotonaldehyde, but not benzene. Kitchens should be properly ventilated to decrease exposure to potentially toxic and carcinogenic fumes produced during Chinese style wok cooking.

## Introduction

Lung cancer is the third most common cancer to occur among women in Singapore, and the second leading cause of cancer death [1]. There were 5520 lung cancer deaths among women in Singapore in the period 2008–2012. The age standardized mortality rate for lung cancer in females in Singapore for that period was 12.2 per 100,000, a figure which has declined only slightly in the past 40 years. Considering that most females in Singapore are non-smokers, these figures are remarkably high and reflect similar incidence data among women in China and some other parts of Southeastern Asia [2]. The relatively high incidence of female lung cancer, particularly adenocarcinoma, in Singapore and other parts of Asia, has been consistently observed and documented [2,3]. Neither tobacco smoking which is relatively rare among women in Asia nor use of solid fuels in poorly ventilated spaces which occurs only in specific regions can explain this unusually pervasive occurrence of adenocarcinoma of the lung in Asian women.

One hypothesis which has been investigated in multiple studies is that fumes from high temperature Chinese style wok cooking may be an etiologic factor for lung cancer. Women do most of the cooking in traditional Chinese households, including in Singapore. The heated oil used in wok cooking, including the common techniques of stir frying and deep frying, produces vapors which contain a variety of potentially mutagenic and carcinogenic compounds [4,5]. Multiple epidemiologic studies have examined this hypothesis with generally, although not exclusively, positive results demonstrating an association between extents of wok cooking and lung cancer risk among non-smoking Asian women [6–16]. In one recent prospective study, there was a significant association between fried meat intake, as a surrogate for high temperature wok cooking, and adenocarcinoma of the lung in non-smoking Chinese women and men in Singapore [17]. The International Agency for Research on Cancer concluded that emissions from high-temperature frying are “probably carcinogenic to humans” [18].

While mutagens and carcinogens have been identified in vapors from high temperature wok cooking, there had been no reports on uptake of these substances by the cooks. Therefore, we carried out a study in which we evaluated uptake of a variety of potential toxicants and carcinogens by 54 non-smoking Singapore women of Chinese ethnicity who regularly did wok cooking at home compared to 50 randomly chosen non-smoking women from among participants in the Singapore Chinese Health Study [19]. The results of that study demonstrated the presence of significantly higher levels of urinary mercapturic acid metabolites of benzene, acrolein, and crotonaldehyde in the cooks compared to the controls. Mercapturic acids are formed by reaction of these compounds or their metabolites with cellular glutathione, followed by metabolic processing and excretion, and are widely used biomarkers for exposure to volatile toxicants and carcinogens [20,21]. Benzene is a carcinogen while acrolein and crotonaldehyde are powerful irritants and DNA-reactive compounds which may play some role in lung carcinogenesis [22,23]. The goal of the study reported here was to further evaluate the relationship between wok cooking and urinary levels of mercapturic acids formed from benzene, acrolein, and crotonaldehyde. The analyses were carried out on urine samples from 328 Chinese women from Singapore who did varying amounts of wok cooking.

## Materials and Methods

### Study Design

We used a cross-sectional study design for the present study. The eligible study participants were healthy Chinese women between the ages of 45 and 74 years residing in Singapore in 2011 who never smoked cigarettes (i.e., less than 100 cigarettes in their lifetime). The estimated sample size of the study was 350 including 100 women who never or rarely did home cooking (≤1 time per week), 150 women who did home cooking 2–6 times per week, and 100 women who did home cooking 7 or more times per week. This sample size would provide a statistical power of 80% to detect an approximately 20–25% difference in urinary mercapturic acids of volatile organic toxicants between the two extreme exposure groups. Study subjects were identified using a combination of purposive sampling and ‘snowballing’. Eligible women, after they provided a written consent, were interviewed in person by a trained interviewer using a structured questionnaire eliciting information on study subjects’ demographics, passive smoking, coffee consumption, and home cooking (including frequency, method, and kitchen ventilation use). Each study participant provided a spot urine sample. All subjects provided written consent for participation. The study was approved by the Institutional Review Boards of the National University of Singapore, the University of Pittsburgh, and the University of Minnsota.

After the collected urine samples were brought to the laboratory at the National University of Singapore, the total volume of the urine and the pH value of the urine were measured and recorded on the study questionnaire. Four aliquots per subject, each containing 4.5 ml of urine, were prepared and stored at −80°C until analysis.

### Biomarker analyses

One of the four aliquots was taken from the repository at the National University of Singapore and shipped in dry ice to the analytical chemistry laboratory at the University of Minnesota. The mercapturic acids of acrolein, crotonaldehyde, and benzene are 3-hydroxypropylmercapturic acid (3-HPMA, abbreviated HPMA in our previous study [19]), 3-hydroxy-1-methylpropylmercapturic acid (HMPMA, abbreviated HBMA previously [19]), and *S*-phenylmercapturic acid (SPMA), respectively. (Also note that the previously reported HBMA values must be multiplied by 5 due to an error in that report; see *Cancer Epidemiology Biomarkers and Prevention* doi: 10.1158/1055-9965.EPI-12-0196). The analyses for 3-HPMA and HMPMA were performed by high throughput liquid chromatography-tandem mass spectrometry using [*N*-acetyl-D_3_]3-HPMA and [*N*-acetyl-D_3_]HMPMA as internal standards, as described [24], while the following modifications were made for analysis of SPMA: 1. [D_5_]SPMA (12.5 ng, Toronto Research Chemicals) was also added to the urine samples as internal standard; 2. Following washing of the 96-well Oasis MAX plates with 0.7 ml of 30% methanol in 2% aqueous formic acid to elute 3-HPMA and HMPMA, the plates were washed with 0.7 ml 50% methanol in 2% aqueous formic acid and this wash was discarded. The plates were then washed with 0.7 ml of 90% methanol in 2% formic acid to collect the fraction containing SPMA and [D_5_]SPMA; 3. The MS transitions monitored were m/z 238.05 → m/z 109.05 for SPMA and *m/z* 243.05 → *m/z* 114.05 for [D_5_]SPMA. Accuracy and precision were as follows: compound, accuracy (%), precision (coefficient of variation); 3-HPMA, 92, 9.1; HMPMA, 97, 11.0; SPMA, 99, 10.

### Creatinine Analysis

Creatinine was analyzed using a colorimetric microplate assay (CRE34-K01) purchased from Eagle Bioscience (http://stores.eaglebio.com/creatinine-microplate-assay-kit).

### Statistical analyses

A total of 350 women were enrolled in the study. Among them, 2 reported having smoked cigarettes based on the in-person interview (ineligible for the study). In addition, the following number of women had missing values on age (n = 1), any one of urinary mercapturic acids (n = 7), or urinary creatinine (n = 12). After excluding ineligible women or those with any missing study variables, the present study included 328 women.

Urinary levels of mercapturic acids 3-HPMA, MHBMA and SPMA were expressed in units of pmol per mg creatinine to correct for varying water contents of individual overnight urine samples. The distributions of these urinary biomarkers were markedly skewed toward high values, which were corrected, to a large extent, by transformation to logarithmic values. Therefore, formal statistical testing was performed on logarithmically transformed values, and geometric (as opposed to arithmetic) means are presented.

One-way analysis of variance (ANOVA) was used to examine the difference in urinary levels of mercapturic acids measured across different categories of demographics (e.g., age, body mass index, level of education, and marital status) and lifestyle factors (e.g., passive smoking exposure and consumption of coffee). Analysis of covariance (ANCOVA) was used to examine the difference in levels of these mercapturic acids by home cooking frequency. The median value of times of home cooking per week within each cooking frequency and the median value of time interval between the last cooking and urine collection were used for linear trend tests with urinary levels of these mercapturic acids. We also examined the difference in levels of these mercapturic acids by other home cooking characteristics only among women with at least 2 or more times of home cooking per week with adjustment for demographic and lifestyle factors as well as cooking frequency.

Statistical analyses were carried out using SAS software version 9.1 (SAS Institute, Cary, NC). All *P*-values reported are two-sided. *P’*s less than 0.05 were considered to be statistically significant.

## Results

The mean age at interview was 59.8 ± 7.7 (SD) years. Demographic characteristics and their possible effects on the mercapturic acids are summarized in **Table 1**. Although we did not obtain information on alcohol consumption, virtually all these nonsmoking Chinese women in Singapore were non-drinkers. There were no significant effects of age, body mass index, level of education, passive smoking exposure, or coffee drinking during the last 3 days on levels of the mercapturic acids. Married women had statistically significantly lower levels of urinary SPMA than women with other marital status. But there was no significant difference in urinary levels of 3-HPMA and HMPMA between the two marital status groups.

**Table 1:** Levels of urinary mercapturic acids by demographic variables in non-smoking Chinese women in Singapore.

Demographic variables	N (%)	Geometric means (95% CI) of mercapturic acids (pmol/mg creatinine)		
		SPMA	3-HPMA	HMPMA
Age, years				
45–54	99 (30.2)	0.50 (0.42–0.58)	1799 (1556–2080)	879 (785–984)
55–64	125 (38.1)	0.48 (0.42–0.56)	2629 (2311–2991)	1134 (1026–1254)
65–74	104 (31.7)	0.56 (0.48–0.64)	2107 (1829–2427)	1023 (916–1142)
P for trend		0.365	0.149	0.068
Body mass index, Kg/m^2^				
<20	45 (13.7)	0.52 (0.40–0.66)	2096 (1682–2612)	1093 (923–1295)
20-<24	141 (43.0)	0.54 (0.46–0.60)	2146 (1896–2430)	969 (881–1066)
24-<28	95 (29.0)	0.52 (0.44–0.60)	2163 (1859–2516)	1067 (950–1199)
28+	47 (14.3)	0.44 (0.36–0.56)	2455 (1980–3044)	991 (840–1170)
P for trend		0.339	0.334	0.913
Level of education				
No	35 (10.7)	0.50 (0.38–0.66)	2316 (1806–2969)	1044 (861–1265)
Primary	112 (34.1)	0.52 (0.44–0.60)	2386 (2077–2742)	1049 (942–1168)
Secondary	140 (42.7)	0.46 (0.40–0.52)	2094 (1849–2371)	1008 (916–1110)
High school or high	41 (12.5)	0.72 (0.56–0.92)	1896 (1507–2385)	937 (784–1119)
P for trend		0.290	0.085	0.321
Marital status				
Married	237 (72.3)	0.48 (0.44–0.54)	2183 (1983–2402)	1003 (931–1080)
Others^a^	91 (27.7)	0.60 (0.52–0.72)	2193 (1879–2560)	1051 (933–1184)
P for difference		0.019	0.959	0.514
Passive smoking				
No	275 (83.8)	0.52 (0.46–0.56)	2152 (1969–2351)	1027 (959–1100)
Yes	53 (16.2)	0.52 (0.42–0.64)	2372 (1938–2903)	962 (823–1124)
P for difference		0.928	0.388	0.450
Coffee drinking during last 3 days				
Not drink	87 (26.5)	0.48 (0.40–0.56)	1995 (1703–2336)	963 (853–1088)
1–3 cups	168 (51.2)	0.52 (0.46–0.58)	2254 (2011–2525)	1049 (961–1145)
4–6 cups	59 (18.0)	0.54 (0.44–0.66)	2335 (1927–2828)	1021 (704–1293)
7+ cups	14 (4.3)	0.62 (0.40–0.94)	2026 (1366–3004)	954 (704–1293)
P for trend		0.228	0.363	0.703

^a^ Includes never married, separated, widowed, and divorced.

The levels of the mercapturic acids were similar to those reported previously[19]. Effects of cooking frequency and the time interval between the last home cooking and urine collection on levels of the mercapturic acids are summarized in **Table 2**. There was a statistically significant effect of cooking frequency on levels of 3-HPMA and HMPMA. Women who cooked more than 7 times per week had a geometric mean of 2600 (95% CI, 2189–3090) pmol/mg creatinine 3-HPMA compared to 1901 (95% CI, 1510–2395) pmol/mg creatinine for those who cooked less than one time per week (P = 0.046). The corresponding values for HMPMA were 1167 (95% CI, 1022–1332) and 894 (95% CI, 749–1067) pmol/mg creatinine (P = 0.027). In each case there were intermediate levels of the biomarkers in the women who cooked 2–6 times per week and the trends were significant, P< 0.018 for 3-HPMA and P< 0.008 for HMPMA. In contrast, there was no effect of cooking frequency on levels of SPMA.

**Table 2:** Levels of urinary mercapturic acids by frequency of home cooking and time interval between last cooking and urine collection in non-smoking Chinese women in Singapore.

	N (%)	Geometric means* (95%CI) of mercapturic acids (pmol/mg creatinine)		
		SPMA	3-HPMA	HMPMA
Times of cooking/week (median)				
≤ 1 (0)	90 (27.4)	0.52 (0.40–0.66)	1901 (1510–2395)^a^	894 (749–1067)^a^
2–6 (5)	143 (43.6)	0.46 (0.40–0.54)	1994 (1733–2295)^a^	920 (826–1024)^a^
7+ (10)	95 (29.0)	0.52 (0.44–0.64)	2600 (2189–3090)^b^	1167 (1022–1332)^b^
P for global test		0.530	0.029	0.009
P for trend		0.652	0.018	0.008
Hours between last cooking and urine collection (median)†				
<12 (2.2)	27 (11.3)	0.40 (0.28–0.54)	2110 (1594–2794)	898 (723–1116)
12-<24 (17.7)	139 (58.4)	0.50 (0.42–0.56)	2224 (1964–2518)	1040 (945–1144)
24-<48 (40.0)	30 (12.6)	0.54 (0.40–0.72)	1992 (1525–2601)	1004 (817–1233)
48+ (80.0)	42 (17.7)	0.50 (0.40–0.66)	2463 (1948–3115)	1077 (899–1291)
P for global test		0.510	0.683	0.608
P for trend		0.416	0.474	0.450

* All geometric means were adjusted for age, body mass index (kg/m^2^), level of education (no, primary, secondary, high school or high), passive smoking (no, yes), marital status (married and others), and coffee consumption (none, 1–3 cups, 4–6 cups, and 7+ cups during last 3 days). For times of cooking per week, the time interval between last cooking and urine collection (<12 hours, 12-<24 hours, 24-<48 hours, 48+ hours or no cooking) was additionally adjusted. For hours between last cooking and urine collection, the frequency of cooking (≤1, 2–6, and 7+/week) was additionally adjusted.

^a,b^ The different superscript letters denote a statistically significant difference in the geometric mean of a given mercapturic acid between the two exposure levels in a pairwise multi-comparison test (P < 0.05).

† Among women who did two or more times of home cooking per week.

Most (90%) of the study participants collected their spot urine between 9 am and 12 noon. The median time interval between the last home cooking and urine collection was 18.8 hours. There was no statistically significant difference in urinary levels of the mercapturic acids across different time intervals between the last home cooking and urine collection after adjustment for the frequency of home cooking (Table 2).

Most Chinese wok cooking is frying that includes stir-frying, pan-frying, and deep-frying methods. Among 238 women who did home cooking 2 or more times per week, 188 (79%) reported using any of the frying cooking methods only, 19 (8%) reported using non-frying cooking methods including boiling and steaming only, and the remaining 31 (13%) used both frying and non-frying cooking methods for their home cooking. Different types of home cooking methods did not have a strong impact on levels of the three urinary mercapturic acids (**Table 3**). Women who did both frying and non-frying cooking had a higher level of 3-HPMA than women who did non-frying cooking only or frying cooking only, and a higher level of HMPMA than women who did frying cooking only. Women who usually used sunflower cooking oil for home cooking had significantly higher levels of urinary SPMA, 3-HPMA and HMPMA than their counterparts using other cooking oils (Table 3). Not using a ventilating hood in the kitchen did not significantly impact levels of the urinary mercapturic acids. Adding cooking wine to food during cooking was associated with a slightly increased urinary level of SPMA (P = 0.083), but did not impact urinary levels of 3-HPMA or HMPMA.

**Table 3:** Levels of urinary mercapturic acids by cooking variables among non-smoking Chinese women in Singapore who did 2 or more times of home cooking per week.

Cooking related variables	N (%)	Geometric means* (95%CI) of mercapturic acids (pmol/mg creatinine)		
		SPMA	3-HPMA	HMPMA
Type of cooking method				
Non-frying cooking only	19 (8.0)	0.72 (0.50–1.06)^a^	1883 (1332–2661)^a^	1170 (896–1528)^a^ ^,^ ^b^
Frying cooking only	188 (79.0)	0.46 (0.40–0.52)^b^	2120 (1868–2406)^a^	950 (861–1047)^a^
Both cooking methods	31 (13.0)	0.48 (0.36–0.66)^a^ ^,^ ^b^	2911 (2202–3848)^b^	1236 (996–1532)^b^
P for global test		0.078	0.068	0.037
Type of cooking oil				
Peanut	75 (31.5)	0.46 (0.36–0.56)^a^	2268 (1881–2736)^a^ ^,^ ^b^	1025 (889–1181)^a^
Canola	49 (20.6)	0.44 (0.34–0.56)^a^	1900 (1523–2370)^a^	829 (702–981)^a^
Sunflower	40 (16.8)	0.64 (0.48–0.84)^b^	2647 (2071–3383)^b^	1329 (1104–1601)^b^
Other (e.g., Olive oil)	74 (31.1)	0.46 (0.38–0.56)^a^	2105 (1740–2547)^a^ ^,^ ^b^	969 (839–1119)^a^
P for global test		0.128	0.224	0.002
Use of hood ventilation in kitchen				
Hood on	53 (22.3)	0.56 (0.44–0.72)	2149 (1736–2661)	938 (796–1105)
Hood off or no hood	185 (77.7)	0.46 (0.40–0.52)	2204 (1937–2506)	1024 (927–1130)
P for difference		0.121	0.766	0.303
Adding cooking wine to food during cooking				
No	212 (89.1)	0.46 (0.40–0.54)	2128 (1887–2400)	998 (909–1095)
Yes	26 (10.9)	0.64 (0.46–0.88)	2741 (2023–3714)	1002 (791–1269)
P for difference		0.083	0.111	0.971

* All geometric means were adjusted for age, body mass index (kg/m^2^), level of education (no, primary, secondary, high school or high), passive smoking (no, yes), marital status (married and others), coffee consumption (none, 1–3 cups, 4–6 cups, and 7+ cups during last 3 days), frequency of cooking (≤1, 2–6, and 7+/week), and the time interval between last cooking and urine collection (<12 hours, 12-<24 hours, 24-<48 hours, 48+ hours or no cooking).

^a,b^ The different superscript letters denote a statistically significant difference in the geometric mean of a given mercapturic acid between the two variables using a pairwise multi-comparison test (P < 0.05).

## Discussion

**Fig 1:**
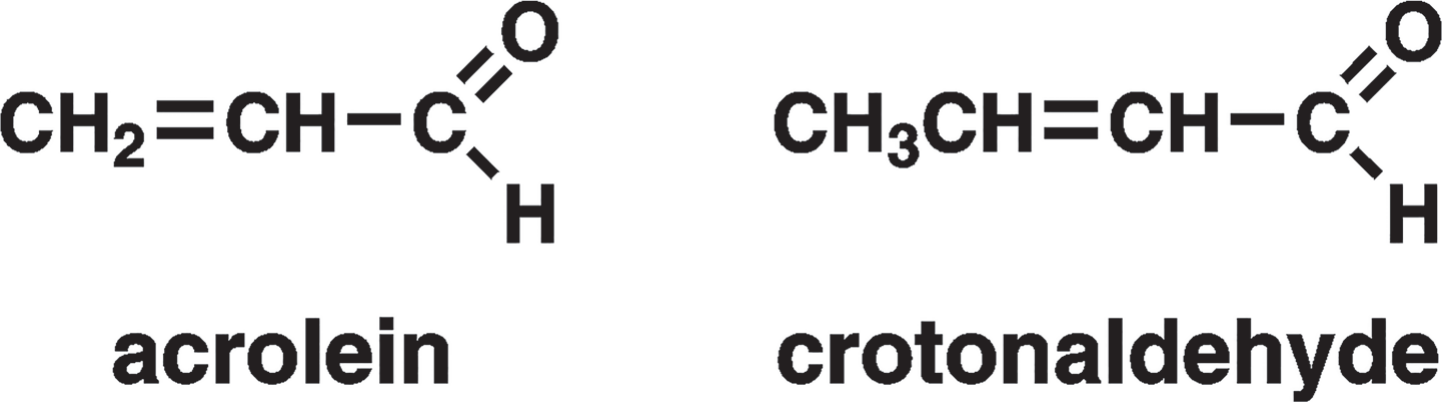
Structures of acrolein and crotonaldehyde.

The results of this study confirm and significantly extend our previous observation of elevated levels of the mercapturic acids of acrolein (3-HPMA) and crotonaldehyde (HMPMA) in the urine of Chinese women from Singapore who regularly cook using a wok [19]. Acrolein and crotonaldehyde are structurally related reactive α,β-unsaturated aldehydes (**Fig. 1**), both of which have been identified in emissions from heated cooking oil. These volatile aldehydes have multiple toxic effects which may be involved in carcinogenesis, although they are not themselves strong carcinogens. Thus, exposure to acrolein and crotonaldehyde might contribute to lung cancer etiology in non-smoking Chinese women. Our results did not however confirm our earlier observation of increased levels of SPMA, the mercapturic acid derived from benzene metabolism, in the urine of these women [19].

Many studies have identified acrolein in emissions from various heated cooking oils [4,25–34]. The type of oil and temperature to which the oil is heated are both important in determining the amount of acrolein produced. For example, Fullana and co-workers demonstrated that canola oil was a more significant source of acrolein than olive oil, at both 180°C and 240°C, with the highest amounts emitted at 240°C [27]. The presence of crotonaldehyde in these vapors has been reported less frequently and generally in lower concentrations than acrolein [27,29,31]. Both acrolein and crotonaldehyde persisted for hours in the indoor environments in which they were generated, and the concentrations could be relatively high. For example, deep-frying with different types of cooking oils resulted in indoor concentrations of acrolein ranging from 26.4–64.5 μg/m^3^, with a half-life of 14–22 h, indicating considerable persistence under conditions of poor ventilation [31]. Crotonaldehyde also persisted under these conditions, with a reported half-life of 20 h [31]. The findings of elevated urinary levels of all three mercapturic acids in women who regularly used sunflower oil for home cooking are intriguing and warrant further investigation.

Acrolein reacts readily with physiologic nucleophiles such as glutathione, ascorbic acid, DNA, and a variety of proteins, and induces multiple toxic effects including in the respiratory tract [34,35]. In humans, it causes intense eye and respiratory irritation which could limit exposure. Multiple inhalation studies of acrolein in laboratory animals have consistently demonstrated irritation, inflammation, cell proliferation, squamous metaplasia, interference with pulmonary function, immunosuppression, weight loss, and other toxic effects [34,35]. Acrolein is toxic to A549 lung cells in vitro and upregulates several acrolein-responsive protein markers [36]. It also activates transient receptor potential ankyrin 1 (TRPA1) channels and causes relaxation of smooth muscle in mouse isolated tracheal segments [37]. Acrolein forms well-characterized adducts in DNA and has a binding pattern in the *p53* tumor suppressor gene similar to the pattern of mutations in this gene found in human lung cancer [38–40]. But carcinogenicity tests of acrolein in mice and rats have yielded negative results [35]. In one study in rats, an increased incidence of urinary bladder papillomas was observed upon intraperitoneal injection of acrolein in combination with dietary uracil [35].

Crotonaldehyde, like acrolein, is a strong irritant to the eye, skin, and respiratory tract in humans, and causes similar effects in laboratory animals [41]. It forms cyclic 1,*N* ^*2*^-deoxyguanosine adducts in DNA, structurally similar to those produced from acrolein [38–40]. Crotonaldehyde produced increased levels of altered liver cell foci, liver damage, and neoplastic nodules in rats treated with the compound in their drinking water [42]. The International Agency for Research on Cancer has evaluated both acrolein and crotonaldehyde as “not classifiable as to their carcinogenicity in humans” [35,41].

While direct evidence for the carcinogenicity of acrolein and crotonaldehyde is weak or lacking, there is no question about their multiple toxic effects and DNA reactivity. It is plausible therefore that these properties of acrolein and crotonaldehyde may play an exacerbating role in carcinogenesis, but the specific pathways involved, if any, remain unclear. Considering these data, it would be prudent to take measures such as improved ventilation to reduce human exposure to acrolein and crotonaldehyde.

The simplest explanation for our results is a direct relationship between frequency of wok cooking and exposure to airborne acrolein and crotonaldehyde. However, it is also possible that the observed increases in mercapturic acid excretion result from exposure via the diet. Acrolein and crotonaldehyde may react with proteins or other nucleophiles in food, and these reactions may be reversible upon consumption of the cooked food, resulting in release of acrolein or crotonaldehyde, which are then detoxified by reaction with glutathione and excreted as mercapturic acids.

We did not observe an effect of frequency of wok cooking on levels of SPMA, the mercapturic acid derived from benzene metabolism, in contrast to the results of our previous study. We have no firm explanation for this result. It is possible however that it could be related to differences in mechanisms of mercapturic acid formation from acrolein, crotonaldehyde, and benzene. Acrolein and crotonaldehyde are highly reactive α,β-unsaturated carbonyl compounds which easily react with glutathione in the absence of catalysis. It is not clear whether catalysis by glutathione-*S*-transferases is necessary for this reaction to occur. Benzene on the other hand is completely unreactive with glutathione. It requires metabolism to benzene oxide, catalyzed mainly by cytochrome P450 2E1, as a prerequisite for formation of SPMA [22]. Most benzene oxide rapidly rearranges to phenol, but some is captured by glutathione with catalysis by glutathione-*S*-transferases leading ultimately to excretion of SPMA in urine. While SPMA is a well validated biomarker of benzene exposure, its more complex route of formation may detract from its overall sensitivity compared to 3-HPMA and HMPMA.

Most Chinese wok cooking is frying, including stir-frying, pan-frying, and deep-frying. Seventy-nine percent of the women in our study reported using exclusively frying methods. As described above, deep-frying could generate substantial amounts of both acrolein and crotonaldehyde [31] whereas there are no reported data on the emission of these volatile toxicants from stir-frying or pan-frying, which use relatively lower oil temperatures than deep-frying. In our questionnaire, we did not separate these different frying methods, and were therefore unable to examine the impact of the different frying methods on urinary levels of the biomarkers. Only 6% of the women prepared their food using non-frying methods, which presumably produce lower quantities of volatile toxicants than frying. In the present study, the mean level of 3-HPMA in urine from women who did non-frying cooking was lower than those who did frying cooking, but the difference was statistically borderline significant due to the small sample size of non-frying cooking. We did observe however that the use of both cooking methods resulted in a significant increase in 3-HPMA and HMPMA compared to frying cooking only, but the basis for this observation is unclear.

This study has certain limitations. Our results were based on a single spot urine sample. We do not know if the results of that analysis are generalizable to a given woman’s typical exposure. We also did not perform air monitoring for volatiles in the kitchens where the women cooked. Multiple urine samples and air monitoring could be considered in future studies, but these activities were not compatible with our budget for the study reported here. A more detailed examination of frying methods could also be incorporated in future investigations. The relatively small sample size, especially with respect to specific cooking methods, might have limited the study power to detect a statistically significant effect.

In summary, the present study demonstrates that women who did frequent wok cooking but did not smoke cigarettes or drink alcoholic beverages had significantly increased levels of urinary 3-HPMA and HMPMA, the respective mercapturic acids of acrolein and crotonaldehyde, and the levels of the biomarkers increased with increasing cooking frequency. These findings confirm and significantly extend our previous observation of elevated levels of these biomarkers in a convenience sample of Chinese women in Singapore with a much smaller sample size. The present data, together with multiple epidemiologic studies, demonstrate the need for preventive measures such as improved ventilation to efficiently remove cooking oil fumes.
